# Paste

**Published:** 1870-01

**Authors:** 


### PASTE

Is often worth more than its weight in gold. How many a time has the reader been impressed with he value of an article in a newspaper, which, if he had but a little paste at hand, he could have attached it to some book for subsequent reference, but having no paste, he lays the paper away, and finally forgets all about it, every now and then, however, feeling that it would be worth more than money if he could lay his hand on that scrap again. As ordinary gum paste m akesprint transparent, that made of flour is better. But a cup of paste should not be allowed to remain in an occupied room, except while using it, as it gives a disagreeable and very unhealthful odor. It may be kept outside on the window-sill, with a brush ready for use. Dissolve a teaspoonful of alum in a quart of warm water. When cold, stir in as much flour as will give it the consistency of thick cream, being particular to beat up all the lumps, then stir in as much powdered resin as will stand on a dime, and throw in half-a-dozen cloves, to give a pleasant odor. Have on the fire a teacupful or more of boiling water, pour the flour mixture into it, stirring it all the time. It will soon be of the consistency of mush. Pour it into an earthen or china vessel; let it cool; lay a cover on, and put it in a cool place. When needed for use, take out a portion, and soften it with warm water.

